# Novel Psychosocial Correlates of COVID-19 Vaccine Hesitancy: Cross-Sectional Survey

**DOI:** 10.2196/45980

**Published:** 2023-09-27

**Authors:** Elizabeth Bacon, Lawrence An, Penny Yang, Sarah Hawley, M Lee Van Horn, Ken Resnicow

**Affiliations:** 1 Center for Health Communications Research Rogel Cancer Center University of Michigan Ann Arbor, MI United States; 2 Division of General Medicine School of Medicine University of Michigan Ann Arbor, MI United States; 3 Veterans Affairs Center for Clinical Management Research Veterans Affairs Ann Arbor Healthcare System Ann Arbor, MI United States; 4 Department of Health Behavior and Health Education University of Michigan School of Public Health Ann Arbor, MI United States; 5 Department of Individual, Family, and Community University of New Mexico Albuquerque, NM United States

**Keywords:** COVID-19 vaccination, health communication, psychological predictors, psychology, public health, conspiracy beliefs, vaccine misinformation, religious beliefs, anti-vaccination beliefs, reactance, dogmatism, political beliefs, health care system distrust, gender roles, gender, online survey, USA, adults, death, illness, virus, psychosocial

## Abstract

**Background:**

Effective COVID-19 vaccines have been available since early 2021 yet many Americans refuse or delayed uptake. As of mid-2022, still around 30% of US adults remain unvaccinated against COVID-19. The majority (81%) of these unvaccinated adults say they will “definitely not” be getting the COVID-19 vaccine. Understanding the determinants of COVID-19 vaccine uptake is critical to reducing death and illness from the virus, as well as to inform future vaccine efforts, such as the more recent bivalent (omicron) booster.

**Objective:**

This study aimed to expand our understanding of psychosocial determinants of COVID-19 vaccine uptake. We focus on both COVID-19–specific factors, such as COVID-19 conspiracy beliefs, as well as more global personality attributes such as dogmatism, reactance, gender roles, political beliefs, and religiosity.

**Methods:**

We conducted a web-based survey in mid-2021 of a representative sample of 1376 adults measuring both COVID-19–specific beliefs and attitudes, as well as global personality attributes. COVID-19 vaccination status is reported at 3 levels: vaccinated; unvaccinated-may-get-it; unvaccinated-hard-no.

**Results:**

Our analyses focused on the correlation of COVID-19 vaccination status with 10 psychosocial attributes: COVID-19-specific conspiracy theory beliefs; COVID-19 vaccine misinformation; COVID-19–related Rapture beliefs; general antivaccination beliefs; trait reactance; trait dogmatism; belief in 2020 election fraud; belief in a QAnon conspiracy; health care system distrust; and identification with traditional gender roles. We used a multivariate analysis of covariance to examine mean differences across vaccine status groups for each of the correlates while holding constant the effects of age, gender, race, income, education, political party, and Evangelicalism. Across the 10 psychosocial correlates, several different response scales were used. To allow for comparison of effects across correlates, measures of effect size were computed by converting correlates to *z* scores and then examining adjusted mean differences in *z* scores between the groups. We found that all 10 psychosocial variables were significantly associated with vaccination status. After general antivaccination beliefs, COVID-19 misinformation beliefs and COVID-19 conspiracy beliefs had the largest effect on vaccine uptake.

**Conclusions:**

The association of these psychosocial factors with COVID-19 vaccine hesitancy may help explain why vaccine uptake has not shifted much among the unvaccinated-hard-no group since vaccines became available. These findings deepen our understanding of those who remain resistant to getting vaccinated and can guide more effective tailored communications to reach them. Health communication professionals may apply lessons learned from countering related beliefs and personality attributes around issues such as climate change and other forms of vaccine hesitancy. For example, using motivational interviewing strategies that are equipped to handle resistance and provide correct information in a delicate manner that avoids reactance.

## Introduction

Over 1.07 million people in the United States have died from COVID-19 and many more have been impacted with long-term morbidity [1]. Effective vaccines have been available since early 2021 yet many Americans refuse or delay uptake. As of mid-2022, around 30% of US adults still remain unvaccinated against COVID-19. The majority (81%) of these unvaccinated adults say they will “definitely not” be getting the COVID-19 vaccine [2]. Understanding the determinants of COVID-19 vaccine uptake is critical to reducing death and illness from the virus, as well as to inform future vaccine efforts, such as the more recent bivalent (omicron) booster.

There is a growing body of literature identifying factors affecting COVID-19 vaccine uptake. However, most studies focus on sociodemographic attributes and political affiliation [2-8]. Some studies have examined more psychological and social factors such as religiosity and COVID-19–specific conspiracy beliefs [9-14]. This study aimed to expand our understanding of psychosocial determinants of COVID-19 vaccine uptake. We focus on both COVID-19–specific factors, such as COVID-19 conspiracy beliefs, as well as more global personality attributes such as dogmatism, reactance, gender roles, political beliefs, and religiosity.

Understanding the relationship of these attributes with COVID-19 vaccination could help design targeted and tailored messaging which could improve the effectiveness of behavioral interventions. We were particularly concerned with understanding what drives those who state they have no intention of ever getting the vaccine, a group also known as “definitely not” or “hard no,” as this group appears to have changed little since the initial availability of the COVID-19 vaccines and remain difficult to persuade [15]. Having such a large unvaccinated subgroup could allow the virus to persist.

This study, fielded in mid-2021, aimed to explore psychosocial factors related to vaccine hesitancy and uptake. This survey was based on an earlier study, done prior to the advent of vaccines, that examined determinants of COVID-19 protective behaviors such as mask-wearing and social distancing [16].

## Methods

### Survey Administration

The survey was fielded from June 15 to 28, 2021. We collected 3225 surveys from age-eligible, consented individuals. To ensure the quality of the respondent data, as we have done in prior studies, we excluded 1792 surveys we deemed as providing invalid responses. This included individuals (n=1720) who completed the full survey in under 10 minutes (the minimum time we considered plausible to complete a valid survey). The mean time for those excluded surveys was 1.5 minutes (SD 2.5 min). We also excluded 236 surveys that were started but not finished. After applying these exclusions, 1433 surveys remained for the present analyses. For the 1433, the mean time to complete the survey was 32.3 (range 10.0 to 604.9, SD 41.1) minutes. There were no significant differences in demographic characteristics (age, gender, race, income, and education) or vaccine status when comparing the included and excluded respondents in chi-square and ANOVA tests.

Surveys were completed through the Qualtrics (XM) web-based platform using a sample provided by Dynata (Dynata) [17]. Dynata’s research panel comprises an opt-in list of over 60 million individuals globally. For this study, we requested a nationally representative sample of 1500 US adults ages 18 years and older.  The sample approximated national rates for age, gender, race, income, and COVID-19 vaccination at the time. The survey was conducted as open enrollment, whereby eligible panel members who logged into the Dynata website were offered a chance to take this survey. Participants received modest compensation (approximately US $1) from Dynata for completing their survey.

### Measures

#### Overview

The survey assessed a range of attitudes and personality attributes that we hypothesized might be related to COVID-19 vaccine uptake based on our prior work and that of others [16]. Our list of potential predictors included both COVID-19–specific constructs as well as more global personality attributes. Informed by the findings of other groups about some of these predictors, as detailed in the Discussion section, we chose to include multiple psychosocial factors together in a single model to examine the relationship with COVID-19 vaccination. The survey consisted of both existing and developed measures. Our team has extensive expertise in measures development [16,18-20]. The full text of each measure described here can be found in Multimedia Appendix 1.

#### COVID-19 Vaccination Status

We assessed COVID-19 vaccination status with a 2-step approach, similar to the classification used in other studies [15]. First, we asked whether someone had received any primary doses of the COVID-19 vaccine. For those who had not, we asked whether they might get it in the future and in what circumstance. From these items, we created the following 3 levels used in all analyses: vaccinated (both doses of 2 dose, 1 dose of 2 dose, or 1 dose of 1 dose vaccine); unvaccinated-may-get-it (which included considering the vaccine and would get it: as soon as you can, wait and see, only if required, do not know when), and unvaccinated-hard-no (definitely would not get the vaccine).

#### COVID-19–Specific Attitudes and Beliefs

*COVID-19 conspiracy beliefs* were measured with 11 items drawn from 2 prior measures [10,16]. Sample items include “The media is making coronavirus seem more dangerous than it really is” and “Bill Gates has put microchips into the COVID-19 vaccine to track people.” Each item was answered along a 5-point continuum, with the first 3 items rated definitely false to definitely true and the last 8 items scaled with do not agree to agree completely. The 2 scales were combined into a composite measure. α was .94 for the 11-item composite scale.

*COVID-19 vaccine misinformation* was measured with 5 items created by the study authors, based on prior surveys and media reports. Each item had response options of true, false, or unsure. Sample items include: “The COVID-19 vaccines have been shown to cause infertility” and “The COVID-19 vaccines can change your DNA.”

We recoded each item into a binary variable where a response of false (ie, believed the factually correct answer) was coded as 0, and a response of true or unsure was coded as 1. We then computed a sum score of these 5 binary items with higher scores indicating more endorsement of COVID-19 misinformation. The score ranged from 0 to 5 and α for the 5 items was .84.

*Religious and Rapture beliefs* were measured using 7 items adapted from our prior measures as well as informed by recent reports [16,21,22]. Each item was answered along a 5-point continuum ranging from strongly disagree to strongly agree. Sample items include: “Prayer will protect me from COVID-19” and “The COVID-19 pandemic is a sign that the rapture is coming.” Responses were averaged to create a mean score with higher values indicating greater endorsement of these beliefs. α for the 7 items was .94.

#### Global Beliefs and Personality Attributes

*General antivaccination beliefs* were measured using 5 items, 4 of which came from a prior scale [23] with the remaining item created for this study. Sample items include: “Although most vaccines appear to be safe, there may be problems that we have not yet discovered” and “I avoid vaccines because I don’t like needles or getting a shot” (new item). Each item was answered along a 5-point continuum with responses ranging from strongly disagree to strongly agree. Responses were averaged to create a mean score with higher values indicating greater vaccine hesitancy. α for the 5 items was .74.

*Trait reactance* was measured with 5 items from the Hong Reactance scale [16]. Each item was answered along a 5-point continuum ranging from strongly disagree to strongly agree. Sample items include: “I become angry when my freedom of choice is restricted” and “Regulations trigger a sense of resistance in me.” Responses were averaged to create a mean score with higher values indicating greater trait reactance. α for the 5 items was .89.

*Political beliefs* were measured using 2 items, based on prior surveys [16]. Each item had response options of true, false, or unsure. The 2 political items, which were combined into a composite scale, were: “Donald Trump actually received more legally valid votes than Joseph Biden” and “There was so much voter fraud that we don't really know who won the election.” We coded a response of false as 0, unsure as 1, and true as 2. Responses were summed to create a total score. A higher score indicates a greater endorsement of *2020 election fraud beliefs* (range 0-4). α for the 2 items was .72.

We also assessed endorsement of QAnon beliefs with a single item: “A group of Satan-worshiping elites who run a child sex ring are trying to control our politics and media.” We recoded this item into a binary variable where a response of false (ie, believed the factually correct answer) was coded as 0 and a response of true or unsure (ie, some level of belief in the misinformation) was coded as 1. Higher scores indicate greater endorsement of QAnon beliefs.

*Health care system distrust* was measured with the 9-item Revised Health Care System Distrust scale [24]. Each item was answered along a 5-point continuum ranging from strongly disagree to strongly agree. Sample items include: “The Health Care System makes too many mistakes” and “The Health Care System puts making money above patients’ needs.” Responses were averaged to create a mean score with higher values indicating greater distrust. α for the 9 items was .81.

*Identification with traditional gender roles* was measured with a single, ordinal item: “People can have a combination of masculine and feminine traits, which may or may not correspond with whether they are male or female. How do you see yourself?” [25]. This item was answered along a 6-point continuum ranging from completely masculine (1) to completely feminine (6).

To create a unidirectional scale, we reverse-coded responses from those who identified as men in the demographic question about gender. For those identifying as female, we left the coding as higher indicating more traditionally feminine. This recoding yielded a scale that measured how strongly someone’s self-reported gender aligned with traditional masculine or feminine gender roles. A higher score indicated a more traditional view of one’s gender role.

*Dogmatism* was measured with 3 items from the 20-item dogmatism scale [26]. Each item was answered along a 7-point continuum ranging from strongly disagree (1) to strongly agree (7). Sample items include: “I am so sure I am right about the important things in life, there is no evidence that could convince me otherwise” and “The things I believe in are so completely true I could never doubt them.” The responses to the 3 items were averaged to create a mean Dogmatism score, with higher values indicating a greater trait of dogmatism. α in our sample was .85.

#### Demographic Variables

*Gender* was initially assessed with 5 categories, male, female, transgender (identify as male), transgender (identify as female), and other. Transgender and other were collapsed leaving 3 categories due to small cell sizes. Gender was dummy-coded with male as the referent compared to all other gender categories for the regression analyses.

*Race or ethnicity* was self-reported as White, Black, Hispanic, Multiracial, and other. Because there were too few respondents who were American Indian or Asian, they were also coded as “other”. Race was dummy-coded with White as the referent compared to all other racial categories for the regression analyses.

*Income* was initially assessed with 9 strata which, for ease of presentation, were collapsed into 3 categories: under US $30,000, US $30,000 to US $74,999, and US $75,000 and above. Income was dummy-coded with US $75,000 and above as the referent compared to all other income categories for the regression analyses.

*Education* was initially assessed with 10 strata which were collapsed into 4 categories to capture the meaningful variation: none through high school or General Educational Development, postsecondary (trade school, some college, or associates), bachelor, and advanced degree (masters, doctoral, or professional). Education was dummy-coded with Advanced Degree as the referent compared to all other education categories for the regression analyses.

*Political party* was assessed with 4 categories: Republican, Democrat, Independent, and something else. Political party was dummy-coded with Republican as the referent for the regression analyses.

*Evangelicalism* was measured with a single item: “Would you describe yourself as a ‘born-again’ or evangelical Christian, or not?” [27]. Response options were “Yes, born again or Evangelical,” “No, not born again or Evangelical,” or “Don’t know.” Responses of no or do not know were collapsed into a single category. Evangelicalism was dummy-coded with yes as the referent for the regression analyses.

The sample size was estimated based on the power needed to detect small to moderate effects of correlates between vaccination groups. The target sample size was at least 250 respondents for each subgroup for which we planned separate analyses, for example, vaccine status, gender, and race.

### Statistical Procedures

#### Overview

First, we examined correlations between determinants. The correlates were moderately related to each other in the hypothesized direction with absolute values of the correlations ranging from .06 to .68 (Table S1 in Multimedia Appendix 1). Given these correlations and the multiple comparisons being performed, we used a multivariate analysis of covariance (MANCOVA) to examine mean differences across vaccine status groups for each of the correlates while holding constant the effects of age, gender, race, income, education, political party, and Evangelicalism.

The hypothesis that, across all comparisons, the covariate-adjusted means of the correlates are the same across vaccine groups was tested with a 22 *df* Wilks lambda *F* test. Given that the null hypothesis was rejected, we then examined the association of each of the 10 correlates with the 3 levels of vaccine status using the 2 *df* F test. When the null hypotheses for this were rejected, we finally examined the contrast between the unvaccinated-hard-no group and each of the other 2 groups.

Across the 10 psychosocial correlates, several different response scales were used. To allow for comparison of effects across correlates, measures of effect size were computed by converting correlates to *z* scores and then examining adjusted mean differences in *z* scores between the groups.

#### Statistical Package

All analyses for this study were performed using SPSS (version 28; IBM Corp).

### Ethical Considerations

This survey project was reviewed and deemed to be exempt (survey without identifying information) by the University of Michigan's institutional review board (HUM00181337). All participants reviewed an informed consent form prior to beginning the survey.

## Results

### Demographics

The sample was 57% (n=804) female, 61% (n=862) White, 14% (n=202) Black, 10% (n=146) Hispanic, and 7% (n=101) multiracial (Table 1). The mean age was 46.8 years old (SD 17.0). About 23% (n=325) of the sample had high school or lower education and 46% (n=405) had at least a bachelor’s degree. Income distribution was about even across the 3 strata. With regard to political parties, 25% (n=357) identified as Republican, 43% (n=605) as Democrat, 25% (n=361) as Independent, and 7% (n=101) as something else. About 60% (n=849) of the sample was vaccinated with at least 1 dose of COVID-19 vaccine, while about 40% (n=584) was fully unvaccinated. Of those unvaccinated, 17% (n=238) were strongly against it, that is, hard no. Our sample was representative of the vaccination status proportions at the time of study as reported by the Centers for Disease Control and Prevention COVID Data Tracker (65% vaccinated and 37% unvaccinated) [28].

**Table 1:** Sample demographics.

			Value, n (%)
**Age (years; mean 46.8, SD 17.0 years)**			
	35 and younger	431 (30.4)	
	36-50	405 (28.5)	
	51-64	322 (22.7)	
	65 and older	261 (18.4)	
**Gender**			
	Male	615 (43.2)	
	Female	804 (56.5)	
	Nonbinary or genderqueer	5 (0.4)	
**Race or ethnicity**			
	White	862 (60.5)	
	Black	202 (14.2)	
	Multiracial	101 (7.1)	
	Hispanic	146 (10.2)	
	Other (includes American Indian, Asian, and others)	114 (8)	
**Income**			
	Under US $30,000	414 (29.1)	
	US $30,000 - US $74,999	522 (36.6)	
	US $75,000 and above	489 (34.3)	
**Education**			
	None through high school or General Educational Development (GED)	325 (22.8)	
	Postsecondary (trade school, some college, or associates)	437 (30.7)	
	Bachelor	405 (28.4)	
	Advanced degree (masters, doctoral, or professional)	257 (18)	
**Evangelicalism**			
	Yes, born again or Evangelical	472 (33.2)	
	No, not born again or Evangelical	782 (55)	
	Do not know	169 (11.9)	
**Political affiliation**			
	Republican	357 (25.1)	
	Democrat	605 (42.5)	
	Independent	361 (25.4)	
	Something else	101 (7.1)	
**Vaccination status**			
	Vaccinated (both doses of 2 dose, 1 dose of 2 dose, or 1 dose of 1 dose vaccine)	849 (59.2)	
	Unvaccinated-may-get-it (get the vaccine as soon as you can, wait and see, only if required, and do not know when)	346 (24.1)	
	Unvaccinated-hard-no (definitely not get the vaccine)	238 (16.6)	

### MANCOVA Analyses

All 10 novel psychosocial variables were significantly associated with vaccination status (Wilks lambda=0.728; Table 2). In each case, the unvaccinated-hard-no group had higher scores compared to the vaccinated group. Higher scores indicate greater endorsement of each correlate. The same was true for the comparison of unvaccinated-hard-no to unvaccinated-may-get-it with the exception of COVID-19–associated Rapture beliefs and identification with traditional gender roles. These attributes were significant when comparing unvaccinated-hard-no to the vaccinated group. Pearson correlations indicated low collinearity among all predictor variables (Table S1 in Multimedia Appendix 1).

**Table 2:** MANCOVA^a^ multivariate analysis of psychosocial correlates by vaccine status.

Correlate and vaccination status					Adjusted mean^b^ (SD)		95% CI		*P* value
**COVID-19–specific attributes**									
	**COVID-19 conspiracy beliefs (range 1-5)**								
		Vaccinated (≥1 dose)		2.53 (0.04)		2.46-2.60		<.001	
		Unvaccinated-may-get-it		2.82 (0.06)		2.71-2.93		<.001	
		Unvaccinated-hard-no		3.50 (0.07)		3.36-3.63		*Reference*	
	**COVID-19 misinformation beliefs (range 0-5)**								
		Vaccinated (≥1 dose)		2.06 (0.06)		1.94-2.17		<.001	
		Unvaccinated-may-get-it		3.25 (0.09)		3.07-3.42		<.001	
		Unvaccinated-hard-no		3.85 (0.11)		3.64-4.06		*Reference*	
	**COVID-19–associated Rapture beliefs (range 1-5)**								
		Vaccinated (≥1 dose)		2.47 (0.04)		2.40-2.54		<.001	
		Unvaccinated-may-get-it		2.61 (0.05)		2.50-2.71		.06	
		Unvaccinated-hard-no		2.77 (0.07)		2.63-2.90		*Reference*	
**General attributes**									
	**Identification with traditional gender role (range 1-6)**								
		Vaccinated (≥1 dose)		5.33 (0.04)		5.26-5.41		.007	
		Unvaccinated-may-get-it		5.56 (0.06)		5.44-5.67		.86	
		Unvaccinated-hard-no		5.54 (0.07)		5.40-5.68		*Reference*	
	**Reactance (range 1-5)**								
		Vaccinated (≥1 dose)		2.63 (0.03)		2.56-2.70		<.001	
		Unvaccinated-may-get-it		2.65 (0.05)		2.55-2.76		<.001	
		Unvaccinated-hard-no		2.97 (0.06)		2.84-3.10		*Reference*	
	**Beliefs that the 2020 election was fraudulent (range 0-4)**								
		Vaccinated (≥1 dose)		1.31 (0.05)		1.21-1.40		<.001	
		Unvaccinated-may-get-it		1.74 (0.08)		1.59-1.89		<.001	
		Unvaccinated-hard-no		2.28 (0.09)		2.10-2.46		*Reference*	
	**General antivaccination beliefs (range 1-5)**								
		Vaccinated (≥1 dose)		2.53 (0.03)		2.47-2.58		<.001	
		Unvaccinated-may-get-it		2.96 (0.04)		2.87-3.04		<.001	
		Unvaccinated-hard-no		3.54 (0.05)		3.43-3.64		*Reference*	
	**Health care system distrust (range 9-45)**								
		Vaccinated (≥1 dose)		23.78 (0.22)		23.35-24.20		<.001	
		Unvaccinated-may-get-it		25.65 (0.34)		24.99-26.31		<.001	
		Unvaccinated-hard-no		29.14 (0.41)		28.34-29.94		*Reference*	
	**Dogmatism (range 1-7)**								
		Vaccinated (≥1 dose)		3.99 (0.05)		3.88-4.09		<.001	
		Unvaccinated-may-get-it		4.09 (0.08)		3.93-4.26		.002	
		Unvaccinated-hard-no		4.48 (0.10)		4.28-4.68		*Reference*	
	**QAnon belief (range 0-1)**								
		Vaccinated (≥1 dose)		0.36 (0.02)		0.33-0.39		<.001	
		Unvaccinated-may-get-it		0.51 (0.03)		0.46-0.56		.001	
		Unvaccinated-hard-no		0.64 (0.03)		0.58-0.70		*Reference*	

^a^MANCOVA: multivariate analysis of covariance.

^b^Model adjusted for age, gender, race, income, education, political party, and Evangelicalism.

### Effect Sizes

**Figure 1:**
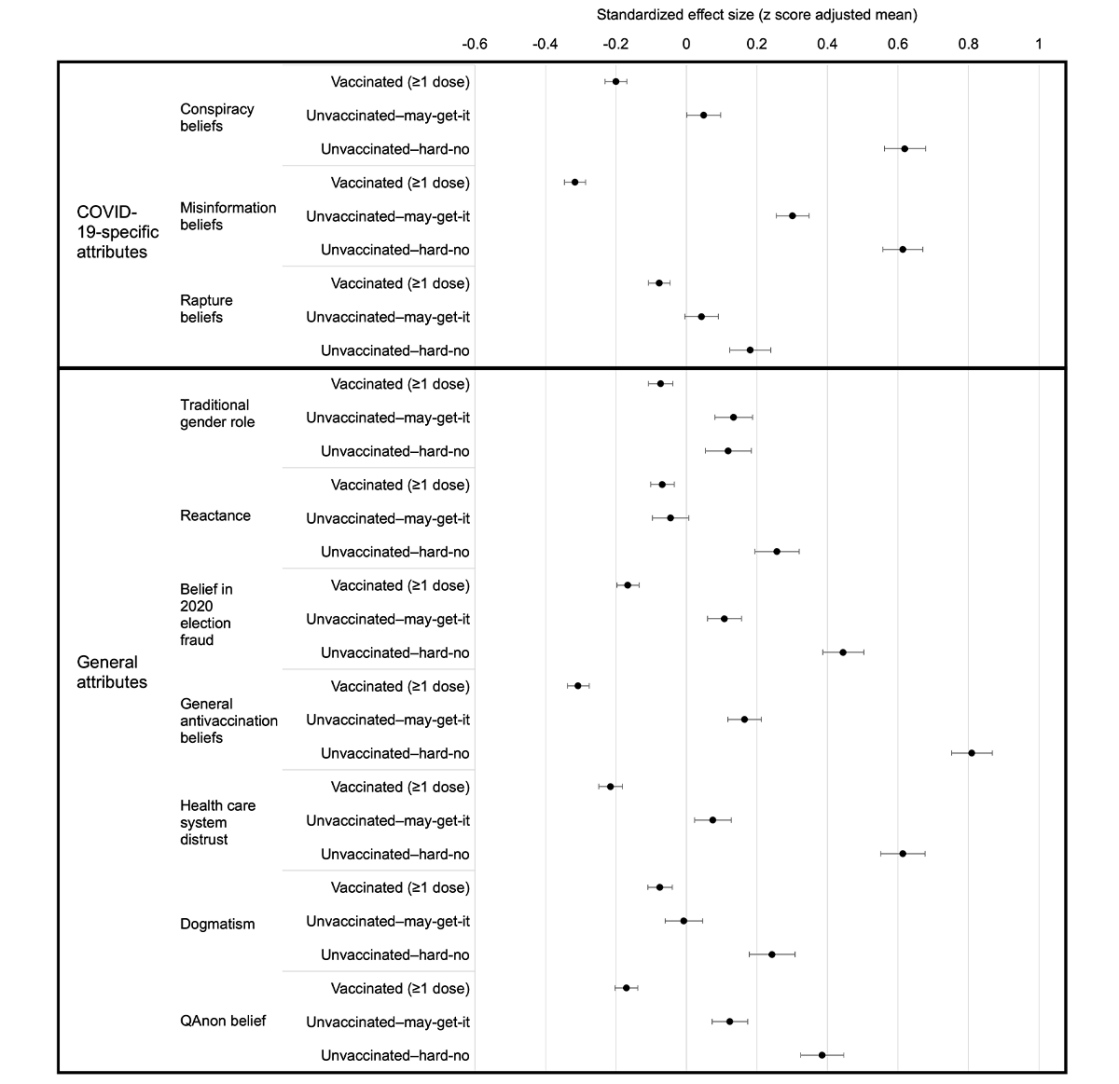
Standardized effect sizes of each psychosocial correlate by vaccine status. Model adjusted for age, gender, race, income, education, political party, and Evangelicalism. Error bars are 2× SE.

To allow comparison of the differences between vaccination groups across the 10 psychosocial correlates, we repeated the MANCOVA analyses shown in Table 2 using the same covariates, but this time with the correlates each standardized using *z* scores to put them on the same scale. This analysis has the same model fit (identical degrees of freedom and Wilk lambda) as the results reported in Table 2, but because each variable is now standardized the different correlates are now on the same scale and can be compared (Figure 1) and the differences between groups are on SD units and can be interpreted as Cohen *d*.

Effect sizes from largest to smallest were general antivaccination beliefs (Cohen *d*=1.12), COVID-19 misinformation beliefs (Cohen *d*=0.93), health care system distrust (Cohen *d*=0.83), COVID-19 conspiracy beliefs (Cohen *d*=0.82), 2020 election fraud beliefs (Cohen *d*=0.61), QAnon belief (Cohen *d*=0.55), reactance (Cohen *d*=0.33), dogmatism (Cohen *d*=0.32), COVID-19–associated Rapture beliefs (Cohen *d*=0.26), and traditional gender role (Cohen *d*=0.19).

## Discussion

### Principal Findings

This study aimed to expand our understanding of the psychosocial drivers of COVID-19 vaccine hesitancy. We examined both COVID-19–specific factors, such as COVID-19 conspiracy beliefs, as well as more global personality attributes such as dogmatism, reactance, gender roles, political beliefs, and religiosity.

We found that all 10 novel psychosocial variables were significantly associated with vaccination status when comparing the vaccinated to unvaccinated-hard-no groups. Effects were adjusted for age, gender, race, income, education, political party, and Evangelicalism. Looking at specific findings, we found that belief in COVID-19 conspiracy theories was higher among those in the unvaccinated-hard-no and unvaccinated-may get-it groups than in the vaccinated group. This is consistent with several prior studies [10-12,29]. Our finding of belief in COVID-19 vaccine misinformation being higher in the unvaccinated-hard-no and unvaccinated-may-get-it groups is also consistent with prior studies [30-33]. After general antivaccination beliefs, COVID-19 misinformation beliefs and COVID-19 conspiracy beliefs had the largest effect on vaccine uptake (Figure 1).

Our findings highlight the role of COVID-19 conspiracy beliefs and misinformation as key drivers of COVID-19 vaccine uptake. Understanding these drivers can help develop tailored vaccine communications including correcting misinformation. Americans are exposed to a great deal of vaccine and COVID-19 misinformation. Further, 1 study found that 73% of participants in the United States had been exposed to at least 1 piece of COVID-19 vaccine misinformation over a 6-month period in 2021, and the more misinformation received negatively impacted vaccine uptake [34]. Another study analyzing COVID-19 information sources on Twitter found an increasing prevalence of and user engagement with unreliable information (ie, conspiracies and misinformation), despite Twitter monitoring this [32]. Exposure to conservative media is also associated with conspiracy beliefs and conservative political ideology, all of which decrease vaccine uptake [35]. Addressing this misinformation may require interventions, such as motivational interviewing, that are equipped to handle resistance and provide correct information in a delicate manner that avoids reactance.

We also found that trait dogmatism was higher among those in both the unvaccinated groups compared to the vaccinated group. This is the first study to examine the relationship between trait dogmatism and COVID-19 vaccine uptake. Other studies have shown that dogmatism is related to both COVID-19 misinformation beliefs and extreme political beliefs, which are also associated with vaccine hesitancy [36-38]. Individuals high on dogmatism may be particularly intractable to information as they do not believe their opinions may be wrong and feel new information is unlikely to alter their beliefs [39].

We also found that a related trait, reactance, was higher in the unvaccinated-hard-no and unvaccinated-may-get-it groups compared to the vaccinated group. This is consistent with prior studies [40,41]. Trait reactance likely influences an individual’s willingness to follow public health or medical recommendations to get the vaccine. Interventions for this group may need to pay particular attention to potential boomerang effects that may entrench the respondent. This group is likely resistant to vaccine appeals from government authorities.

Our results showing greater endorsement of COVID-19–associated Rapture beliefs among unvaccinated-hard-no and unvaccinated-may-get-it groups compared to vaccinated is similar to other groups [9,13,42]. This finding adds nuance to our understanding of the role of religious beliefs in vaccine hesitancy. It is known that religious beliefs play an important role in vaccine hesitancy with about 10% of people in the United States believing that receiving the COVID-19 vaccine conflicts with their faith, including the majority of Evangelicals [43]. However, we found that the effect of COVID-19–associated Rapture beliefs is separate from the effect of Evangelicalism. Addressing religious concerns about vaccination may require messaging highly tailored to different faith systems.

Consistent with other reports, we found that our measures of far-right political ideology (ie, the 2020 election and QAnon beliefs) were higher in unvaccinated-hard-no and unvaccinated-may-get-it groups compared to vaccinated [40-42,44]. This may be related to exposure to conservative, right-wing media [35].

We found that people in the unvaccinated-hard-no group viewed themselves as having more traditional gender roles than those who were vaccinated. Though other studies have looked at gender-based differences in vaccination, ours is novel in reporting the effect of self-identified traditional gender roles on vaccination status. Gender norms may be contributing to women’s hesitancy due to misinformation and access [45,46].

Our findings that both antivaccination beliefs and health care system distrust were higher in unvaccinated-hard-no and unvaccinated-may-get-it groups compared to the vaccinated group aligns with other COVID-19 vaccination studies [47,48].

### Limitations and Future Studies

We excluded over 50% (n=1792) of eligible respondents due to invalid responses. This has the potential to limit generalizability if there were underlying differences in those we excluded compared to those we included. We found no significant differences when comparing these groups for demographic characteristics or vaccination status, but it is possible there are other underlying attributes that differ. Our data were cross-sectional, limiting directional inference. It is possible, for example, that behaviors might influence attitudes rather than the inverse. Longitudinal studies are needed to determine the predictive validity of the correlates we identified. The sample was accrued entirely on the internet which introduces several potential sampling and response biases [49,50]. For example, our sample had a greater percentage of females and Democrats than the US population. Sample bias poses a lower threat to the validity of our findings as we were primarily interested in exploring the association between variables rather than establishing the true prevalence of the attitudes and behaviors under study. There are other potential personality and attitudinal predictors of COVID-19 vaccine uptake we did not measure, including general conspiracy orientation (we measured only COVID-19 conspiracy beliefs), mistrust of government, mistrust of science, paranoia, autonomy needs, hostility, intelligence, pessimism, and media literacy. How these constructs may relate to vaccine uptake merits investigation. We did not measure structural factors that may also be impacting vaccine uptake, such as access to and availability of vaccines. Future studies are needed to replicate and extend our findings, including by examination of how other psychosocial and demographic factors may interact with the 10 correlates we studied. Additionally, work is needed to determine how best to tailor messages, both on the group and individual level, based on these constructs. Finally, our study was conducted before the bivalent (omicron) booster was available. Studies are needed to determine whether the predictors of initial dose uptake we identified herein operate similarly for the uptake of the bivalent booster.

### Intervention Implications

Designing communications to encourage the adoption of COVID-19 vaccines for those with high dogmatism and reactance as well as certain political and religious beliefs poses significant challenges. These individuals may be particularly immune to accepting new information and yielding their beliefs. The persistence of these beliefs among the unvaccinated-hard-no may in part be due to having roots in deeper psychological attributes, such as paranoia or hostility, which we did not measure. Our findings may help explain why vaccine uptake has not shifted much among this segment since vaccines became available [2].

Health communication professionals may apply lessons learned from countering related beliefs and personality attributes around issues such as climate change and other forms of vaccine hesitancy. For example, using motivational interviewing strategies in both public messaging and clinical encounters may be worth testing.

Interestingly, the predictors with the largest effects on vaccine uptake were mostly states, that is, COVID-19–specific beliefs, while the smaller effect sizes were mostly found for traits. States may be more amenable to change with effective counter-messaging and one-on-one counseling. Further, 1 lesson learned from countering other antivaccination beliefs is that simply providing corrective information may not only be ineffective but could instigate further reactance, leading to entrenchment of antivaccine attitudes [51]. Though trait reactance itself is unlikely to be changed, messages designed for individuals prone to reactance could be tailored to the attribute by minimizing controlling language (eg, you must, or you have to) and emphasizing individual autonomy to receive the vaccine [52,53].

## Appendix

Multimedia Appendix 1 Survey items and Pearson correlation table.

## Abbreviations

MANCOVA: multivariate analysis of covariance

## Data Availability

Anonymized data is available from the first author upon request.
