# Supplementary material for: Novel Psychosocial Correlates of COVID-19 Vaccine Hesitancy: Cross-Sectional Survey
Source: JMIR Form Res. 2023 Sep 27;7:e45980. doi: 10.2196/45980 (PMC10538360; doi:10.2196/45980)
Supplement: Multimedia Appendix 1 [file formative_v7i1e45980_app1.docx]

**Multimedia Appendix 1.** Survey Items

### COVID-19 Vaccination Status

Have you personally received the COVID-19 vaccine, or not?

- Yes, got one-dose vaccine
- Yes, got first dose of two-dose vaccine
- Yes, got both doses of a two-dose vaccine
- No, have not gotten the vaccine
- Don't know

[If No or Don’t know to previous question]

How are you currently feeling about getting the COVID vaccine? Would you…

- Definitely get it
- Probably get it
- Probably not get it
- Definitely not get it

### COVID-19-specific attitudes and beliefs

*COVID-19 conspiracy beliefs*

Below are things that some people might believe. Please indicate whether you personally think each statement is true or false.

*Response options:* Each item was answered along a 5-point continuum ranging from Definitely false to Definitely true.

1. The real truth about coronavirus is being kept from the public.
2. People in power are using coronavirus as an excuse to monitor and control the public.
3. The media is making coronavirus seem more dangerous that it really is.

Below are things that some people might believe about COVID-19 and the virus that causes it. Please indicate how much you personally agree with each statement.

*Response options:* Each item was answered along a 5-point continuum ranging from Do not agree to Agree completely.

1. The government is misleading the public about the cause of the virus.
2. I’m skeptical about the official explanation about the cause of the virus.
3. I don’t trust the information about the virus from scientific experts.
4. The virus is manmade.
5. The number of cases and deaths from COVID-19 is being greatly exaggerated.
6. Bill Gates has put microchips into the COVID-19 vaccine to track people.
7. This spread of the virus is a deliberate attempt by a group of powerful people to gain advantage or control.
8. The virus leaked from a lab in Wuhan, China.

*COVID-19 vaccine misinformation*

Do you believe the following statements about the COVID-19 vaccine are true or false or are you unsure?

*Response Options:* True; False; Unsure

1. The COVID-19 vaccines contain fetal cells.
2. The COVID-19 vaccines have been shown to cause infertility.
3. The COVID-19 vaccines can change your DNA.
4. You should not get the vaccine if you have already had COVID-19.
5. You can get COVID-19 from the vaccine.

*Religious and Rapture beliefs*

There are different views on the relationship between religious beliefs and COVID-19. How much do you agree or disagree with the following statements? Please answer even if you’re not religious.

*Response options:* Each item was answered along a 5-point continuum ranging from Strongly disagree to Strongly agree.

1. The COVID-19 vaccine is “the mark of the Beast.”
2. I don’t need the COVID-19 vaccine because God will protect me.
3. Prayer will protect me from COVID-19.
4. God will save us from COVID-19.
5. The COVID-19 pandemic is a sign that the apocalypse is coming.
6. The COVID-19 pandemic is a sign that Jesus will soon be returning.
7. The COVID-19 pandemic is a sign that the rapture is coming.

### Global beliefs and personality attributes

*General anti-vaccination beliefs*

The following statements are about vaccines in general, NOT specifically about the COVID-19 vaccine. Please tell us how much you disagree or agree with the following statements about vaccines in general.

*Response options:* Each item was answered along a 5-point continuum ranging from Strongly disagree to Strongly agree.

1. I feel safe after being vaccinated. (REVERSE CODED)
2. Although most vaccines appear to be safe, there may be problems that we have not yet discovered.
3. Vaccination programs are a big con.
4. Natural exposure to viruses and germs gives the safest protection.
5. I avoid vaccines because I don’t like needles or getting a shot.

*Trait reactance*

How much to do you agree or disagree with the following statements?

*Response options:* Each item was answered along a 5-point continuum ranging from Strongly disagree to Strongly agree.

1. I become angry when my freedom of choice is restricted.
2. Regulations trigger a sense of resistance in me.
3. When something is prohibited, I usually think, “That’s exactly what I am going to do.”
4. It disappoints me to see others submitting to society’s standards and rules.
5. Advice and recommendations usually induce me to do just the opposite.

*Political beliefs*

Below are things that some people might believe. Please indicate whether you personally think each statement is true or false.

*Response options:* True, False, or Unsure.

1. A group of Satan-worshiping elites who run a child sex ring are trying to control our politics and media.
2. Donald Trump actually received more legally valid votes than Joseph Biden.
3. There was so much voter fraud that we don't really know who won the election.

*Health care system distrust*

The next questions are about your opinion of the health care system in general. When we refer to the health care system, we mean hospitals, health insurance companies, and medical research. For each statement below, please check how strongly you agree or disagree.

*Response Options:* Each item was answered along a 5-point Likert scale from Strongly disagree to Strongly agree.

1. The Health Care System does its best to make patients’ health better.
2. The Health Care System covers up its mistakes.
3. Patients receive high quality medical care from the Health Care System.
4. The Health Care System makes too many mistakes.
5. The Health Care System puts making money above patients’ needs.
6. The Health Care System gives excellent medical care.
7. Patients get the same medical treatment from the Health Care System, no matter what the patient’s race or ethnicity.
8. The Health Care System lies to make money.
9. The Health Care System experiments on patients without them knowing.

*Identification with traditional gender roles*

People can have a combination of masculine and feminine traits, which may or may not correspond with whether they are male or female.

How do you see yourself? Would you say that you see yourself as:

- Completely masculine
- Mostly masculine
- Slightly masculine
- Slightly feminine
- Mostly feminine
- Completely feminine

*Dogmatism*

How much do you agree or disagree with the following statements?

*Response Options:* 7-point Likert Strongly disagree (1) – Strongly agree (7)

1. I am a long way from reaching final conclusions about the central issues in life. (REVERSE CODED)
2. I am so sure I am right about the important things in life, there is no evidence that could convince me otherwise.
3. The people who disagree with me may well turn out to be right.  (REVERSE CODED)
4. Twenty years from now, some of my opinions about the important things in life will probably have changed. (REVERSE CODED)
5. The things I believe in are so completely true I could never doubt them.
6. There are no discoveries or facts that could possibly make me change my mind about the things that matter most in life.

**Table S1.** Pearson correlations among dependent variables.

Correlations between predictors ranged from -0.22 to + 0.68. These correlations indicate low collinearity among predictor variables (Table 2). For all correlations *P < .001,* except where noted.

|  | | COVID-19-specific attributes^a^ | | | General attributes^b^ | | | | | | |
| --- | --- | --- | --- | --- | --- | --- | --- | --- | --- | --- | --- |
|  |  | Conspiracy | Misinfo | Rapture | Gender Role | Reactance | 2020 Election | Anti-Vaccine | Healthcare Distrust | Dogmatism | QAnon |
| COVID-19-specific attributes | Conspiracy | 1.00 | .60 | .58 | -.12 | .60 | .59 | .68 | .47 | .44 | .41 |
|  | Misinfo | .60 | 1.00 | .54 | -.13 | .49 | .42 | .61 | .41 | .33 | .50 |
|  | Rapture | .58 | .54 | 1.00 | -.14 | .51 | .38 | .52 | .25 | .45 | .42 |
| General attributes | Gender Role | -.12 | -.13 | -.14 | 1.00 | -.22 | -.06^c^ | -.14 | -.15 | -.10 | -.07^d^ |
|  | Reactance | .60 | .49 | .51 | -.22 | 1.00 | .37 | .54 | .39 | .53 | .33 |
|  | 2020 Election | .59 | .42 | .38 | -.06^c^ | .37 | 1.00 | .44 | .23 | .26 | .39 |
|  | Anti-Vaccine | .68 | .61 | .52 | -.14 | .54 | .44 | 1.00 | .52 | .41 | .40 |
|  | Healthcare Distrust | .47 | .41 | .25 | -.15 | .39 | .23 | .52 | 1.00 | .16 | .26 |
|  | Dogmatism | .44 | .33 | .45 | -.10 | .53 | .26 | .41 | .16 | 1.00 | .23 |
|  | QAnon | .41 | .50 | .42 | -.07^d^ | .33 | .39 | .40 | .26 | .23 | 1.00 |

^a^ Full variable labels (left to right): COVID-19 conspiracy beliefs, COVID-19 vaccine misinformation, COVID-19 religious and Rapture beliefs.

^b^ Full variable labels (left to right): Identification with traditional gender roles, Trait reactance, 2020 election fraud belief score, General anti-vaccine beliefs, Healthcare system distrust, Dogmatism, QAnon belief (“A group of Satan-worshiping elites who run a child sex ring are trying to control our politics and media.”).

^c^ *P*=0.025

^d^ *P*=0.011
